# The bacteriovorous ciliate *Uronema marinum* as a natural biological control agent against *Vibrio* infections in bivalve hatcheries: a sustainable alternative to antibiotics

**DOI:** 10.3389/fmicb.2026.1806526

**Published:** 2026-04-10

**Authors:** Alejandro Cés, Antía Villada, Jesús Lamas, Susana Novoa, Justa Ojea, Susana Prado, Juan Luis Barja, José Manuel Leiro

**Affiliations:** 1 Parasitology Unit, Department of Microbiology and Parasitology, Institute of Aquatic Research for Global Health (iARCUS), Galician Research Foundation in Health Sciences (IDIS), University of Santiago de Compostela, Santiago de Compostela, Spain; 2 Centro de Cultivos Mariños de Ribadeo (CIMA), Xunta de Galicia, Ribadeo, Lugo, Spain; 3 Cell Biology Unit, Department of Functional Biology, Institute of Aquatic Research for Global Health (iARCUS), Galician Research Foundation in Health Sciences (IDIS), University of Santiago de Compostela, Santiago de Compostela, Spain; 4 Microbiology Unit, Department of Microbiology and Parasitology, Institute of Aquatic Research for Global Health (iARCUS), University of Santiago de Compostela, Santiago de Compostela, Spain

**Keywords:** antibiotic alternatives, aquaculture, bacteriovorous ciliate, biological control, bivalve larvae, *Philasterides dicentrarchi*, *Uronema marinum*, *Vibrio* spp.

## Abstract

**Introduction:**

Bacterial infections caused by *Vibrio spp*. are among the main factors limiting the survival of bivalve larvae in hatcheries, and the overuse of antibiotics has promoted resistance and environmental concerns. This study evaluated the free-living marine ciliate *Uronema marinum* as a natural biological control agent against *Vibrio* pathogens affecting clam larvae.

**Methods:**

The ciliate was identified by morphology and 18S rRNA gene sequencing. Its cytopathogenicity was assessed in EPC cell monolayers using *Philasterides dicentrarchi* as a pathogenic control. Bacteriovory was evaluated by growth assays with several virulent *Vibrio* species, by comparing active versus heat-inactivated bacteria, and by fluorescence/Nomarski microscopy using FITC-labeled bacteria. Protection assays were performed in larvae of *Ruditapes philippinarum* and *R. decussatus* challenged with *Vibrio alginolyticus* or *P. dicentrarchi*, with or without *U. marinum* or gentamicin.

**Results:**

*Uronema marinum* was confirmed as a non-cytopathogenic, strictly bacteriovorous ciliate. It proliferated efficiently on several virulent Vibrio species, reaching densities above 4 × 10^4^ ciliates mL^−1^, whereas no growth occurred with heat-inactivated bacteria. Microscopy demonstrated active and selective ingestion of FITC-labeled bacteria and exclusion of microalgal cells. In infection assays, co-incubation with *U. marinum* significantly improved larval survival after challenge with *V. alginolyticus* or *P. dicentrarchi*, maintaining protection comparable to gentamicin. The ciliate also protected larvae across bacterial concentrations of 10^3^–10^5^ CFU mL^−1^.

**Discussion:**

These results provide the first direct experimental evidence that a bacteriovorous ciliate can reduce *Vibrio*-induced mortality in bivalve larvae through selective bacteriovory. The non-pathogenic nature, trophic selectivity, and protective efficacy of *U. marinum* support its potential as a sustainable and environmentally safe alternative to antibiotics in bivalve hatcheries.

## 1 Introduction

Bivalve hatcheries are essential for aquaculture production and the restoration of natural molluscan populations. However, larval mortality caused by bacterial diseases—particularly those associated with *Vibrio* species—remains one of the main bottlenecks limiting productivity and economic sustainability (Dubert et al., 2016, 2017; Prado et al., 2014). Outbreaks of vibriosis in larvae of *Ruditapes, Crassostrea*, and *Mytilus* genera are frequently reported in hatcheries worldwide and are mainly attributed to virulent species such as *Vibrio alginolyticus, V. splendidus, V. pectenicida, V. ostreicida*, and *V. neptunius* (Beaz-Hidalgo et al., 2010).

The conventional management of these infections relies heavily on antibiotic use—particularly aminoglycosides such as gentamicin—to reduce bacterial loads in larval rearing systems (Dubert et al., 2017). However, the indiscriminate application of antibiotics has led to the emergence of resistant bacterial strains, disruption of beneficial microbiota, and accumulation of residues in coastal ecosystems (Cabello et al., 2013). The excessive use of antibiotics in aquaculture has resulted in global dissemination of resistance genes and environmental accumulation of antibiotic residues in surrounding aquatic environments, potentially promoting antimicrobial resistance and altering natural microbial communities (Van Boeckel et al., 2015; Wang et al., 2024). These concerns have intensified the search for sustainable alternatives to chemotherapeutics capable of controlling bacterial diseases without compromising environmental safety or larval health.

Recent advances in aquaculture microbiology have explored a variety of biological control strategies, including probiotics, microalgae, and quorum-quenching bacteria that inhibit pathogen virulence rather than directly killing them (Defoirdt et al., 2011; Pérez-Sánchez et al., 2018; Defoirdt et al., 2011). While bacteriophages and probiotic bacteria have shown promise, their efficacy is often limited by strain specificity, phage resistance, or instability under hatchery conditions (Defoirdt et al., 2011; Kalatzis et al., 2018; Pérez-Sánchez et al., 2018). Therefore, novel biological approaches targeting pathogenic bacteria through ecological rather than antimicrobial mechanisms are urgently needed.

In this context, bacteriovorous protists such as ciliates represent an entirely different and largely unexplored avenue for biological control. These microeukaryotes are natural components of marine microbial food webs and play an essential role in regulating bacterial abundance through continuous grazing (Sherr and Sherr, 2002). Their ability to consume a broad range of bacterial prey offers a potential means to reduce the density of opportunistic pathogens, thereby promoting microbiological balance in rearing environments.

The marine scuticociliate *Uronema marinum* is a free-living protist widely distributed in coastal habitats, where it has been described as an efficient consumer of bacterial populations (Berk et al., 1976; Dragesco and Dragesco-Kernéis, 1986; Song et al., 2009; Sherr and Sherr, 2002). Despite its phylogenetic proximity to the fish pathogen *Philasterides dicentrarchi, U. marinum* is non-parasitic and exclusively bacteriovorous (Iglesias et al., 2001; Paramá et al., 2006). Its presence in hatchery microcosms suggests that it may naturally contribute to the control of opportunistic bacteria, including pathogenic *Vibrio* spp., though this ecological role has never been experimentally demonstrated.

This study provides the first experimental evidence that a bacteriovorous ciliate can act as a natural biological control agent against *Vibrio*-induced infections in bivalve larvae. Specifically, we (i) confirmed the non-pathogenic character of *U. marinum* through cytopathogenicity assays, (ii) characterized its bacteriovorous capacity against multiple *Vibrio* species (*V. alginolyticus, V. splendidus, V. pectenicida, V. ostreicida*, and *V. neptunius*), (iii) demonstrated the requirement of live bacteria for ciliate proliferation, and (iv) evaluated its protective effect on clam larvae challenged with pathogenic bacteria or ciliates, comparing its performance with gentamicin.

Our results reveal a novel ecological mechanism for pathogen control based on predator–prey interactions rather than antimicrobial activity, highlighting *U. marinum* as a promising, environmentally friendly alternative to antibiotics in bivalve hatcheries.

## 2 Materials and methods

### 2.1 Larval culture of *Ruditapes philippinarum, Ruditapes decussatus*, and *Venerupis corrugata*

Adult broodstock of *R. philippinarum, R. decussatus*, and *V. corrugata* were maintained at the CIMA Ribadeo hatchery (Galicia, NW Spain) in flow-through seawater and conditioned under controlled temperature and photoperiod. Seawater used throughout spawning and larval rearing was sand-filtered and further filtered to 1 μm and sterilized by UV. Temperature, salinity, and dissolved oxygen were monitored daily and maintained within the species-appropriate ranges typical for clam hatchery production (18–22 °C; 30–35 PSU; dissolved oxygen >6 mg L^−1^), unless otherwise specified.

Spawning was induced by thermal stimulation (alternating increases and decreases of 3–5 °C) and gentle desiccation, and gametes were collected separately to avoid polyspermy. Eggs were fertilized using diluted sperm to achieve an approximate sperm-to-egg ratio of 5–10:1. After 10–20 min, embryos were rinsed through appropriate mesh to remove excess sperm and debris and incubated until the D-larval stage in gently aerated tanks.

Newly formed D-larvae were stocked in conical-bottom larval tanks (200–1,000 L) at an initial density of 5–10 larvae mL^−1^ with continuous mild aeration. Larvae were fed daily with a mixed microalgal diet, typically including *Isochrysis galbana* (T-ISO) and *Chaetoceros* spp. (with *Tetraselmis* spp. added when available), starting at 5–10 × 10^3^ cells mL^−1^ day^−1^ and progressively increasing with larval size up to 50–80 × 10^3^ cells mL^−1^ day^−1^. Partial water exchanges (30%−50%) were performed daily (or every other day depending on tank condition), and larval tanks were siphoned to remove settled material and dead larvae. Larval growth and survival were assessed at regular intervals by microscopic examination and volumetric counts. To reduce size heterogeneity, larvae were graded every 3–5 days using a cascade of sieves (typically 40–100 μm, adjusted to larval size) and returned to clean tanks at adjusted densities.

The microalgal culture *(I, galbana)* was cultured at the hatchery facilities of the Centro de Cultivos Mariños de Ribadeo (CIMA-Ribadeo) following standard microalgal production protocols routinely used for bivalve larval nutrition. Algal stocks were maintained under axenic or low-bacterial conditions in filtered (1 μm) and UV-sterilized seawater enriched with f/2 nutrients. Cultures were grown at 18–22 °C under continuous aeration and a 16:8 h light:dark photoperiod, using cool-white fluorescent illumination at approximately 100–150 μmol photons m^−2^ s^−1^.

Starter cultures were progressively scaled up from laboratory flasks to larger culture volumes according to hatchery demand. Algal growth was monitored daily by cell counts using light microscopy, and cultures were harvested during the exponential growth phase to ensure optimal nutritional quality. Prior to use, algal suspensions were gently homogenized and directly supplied fresh to larval rearing tanks, either alone or as part of mixed diets with other microalgal species, depending on the experimental design.

### 2.2 Isolation and culture of ciliates

Ciliates were isolated from 50 mL seawater samples containing moribund *Donax trunculus* larvae collected from the hatchery at Centro de Cultivos Marinos de Ribadeo (CIMA, Lugo, Spain). Samples were distributed in 12-well plates and maintained at 21 °C. Wells containing dense ciliate populations were subcloned by limiting dilution into sterile seawater. Monoxenic cultures were established using *E. coli* DH5α (108 bacteria mL^−1^) as a food source in 24-well plates, followed by scale-up to 25–175 cm^2^ flasks for mass production. The *Escherichia coli* strain DH5α used as the bacterial food source in monoxenic cultures of *U. marinum* is a genetically modified, non-pathogenic laboratory strain widely employed for recombinant DNA work and heterotrophic maintenance of protists. It lacks functional virulence factors and antibiotic resistance determinants and has several mutations that impair recombination and endonuclease activity, such as *recA1* and *endA1* (Hanahan, 1983; Grant et al., 1990; Raleigh et al., 1988). These characteristics make DH5α biologically safe and suitable for use in microbiological and protozoological experimental systems.

### 2.3 Identification of ciliates and bacteria

Morphological and molecular identification of the isolated ciliates was carried out to confirm species identity. The infraciliature of the ciliates was visualized following the silver carbonate method described by Budiño et al. (2011). Cells were fixed in Bouin's solution (10–15 min), washed in 70% ethanol, and air-dried on gelatin–chrome alum–coated slides. A freshly prepared silver carbonate suspension (2% AgNO_3_ mixed with saturated Na_2_CO_3_) was applied to the dried smear, and slides were gently heated at 58–60 °C until kineties became visible. The reaction was stopped with 5% sodium thiosulfate, and slides were rinsed with distilled water, air-dried, mounted in Entellan^®^, and examined under bright-field microscopy. Morphological characterization included examination of cell size, shape, and the structure of the oral ciliature using ammoniacal silver carbonate staining.

Genomic DNA was extracted using the **GeneJET Genomic DNA Purification Kit** (Thermo Scientific, Madrid, Spain) following the manufacturer's instructions. A fragment of the small subunit ribosomal RNA gene (18S rRNA; nt 1,389–1,510) was amplified by PCR and sequenced (Sanger method). The resulting sequences were analyzed using the BLAST tool against the NCBI database. The strain of *P. dicentrarchi* used as a pathogenic control was previously isolated from turbot (*Scophthalmus maximus*) suffering scuticociliatosis and maintained in our culture collection. Its identity was confirmed by morphological analysis (presence of two contractile vacuoles, distinct peristomial field, and cytostome morphology) and by molecular sequencing of the 18S rRNA gene, which showed 100% similarity with reference sequences of *P. dicentrarchi* (GenBank accession numbers AY541683, FJ545689) (Iglesias et al., 2001; Paramá et al., 2006, 2003). Bacterial strains used in infection assays corresponded to well-characterized isolates from bivalve hatcheries, identified and coded according to Toranzo and Barja (1990), Prado et al. (2014), and Beaz-Hidalgo et al. (2010): *Vibrio alginolyticus* (strain CECT 521), *Vibrio splendidus* (strain DMC-1), *Vibrio pectenicida* (strain DMC-12), *Vibrio ostreicida* (strain PP-2), and *Vibrio neptunius* (strain PP-3). Identification was confirmed by colony morphology, biochemical profiling (API 20E system, bioMérieux), and sequencing of the 16S rRNA gene using universal primers 27F and 1492R (Lane, 1991; Weisburg et al., 1991). All strains are recognized pathogens of bivalve larvae and are routinely used as virulent reference isolates in experimental challenge assays.

### 2.4 Cytopathogenicity assay

**Figure 1:**
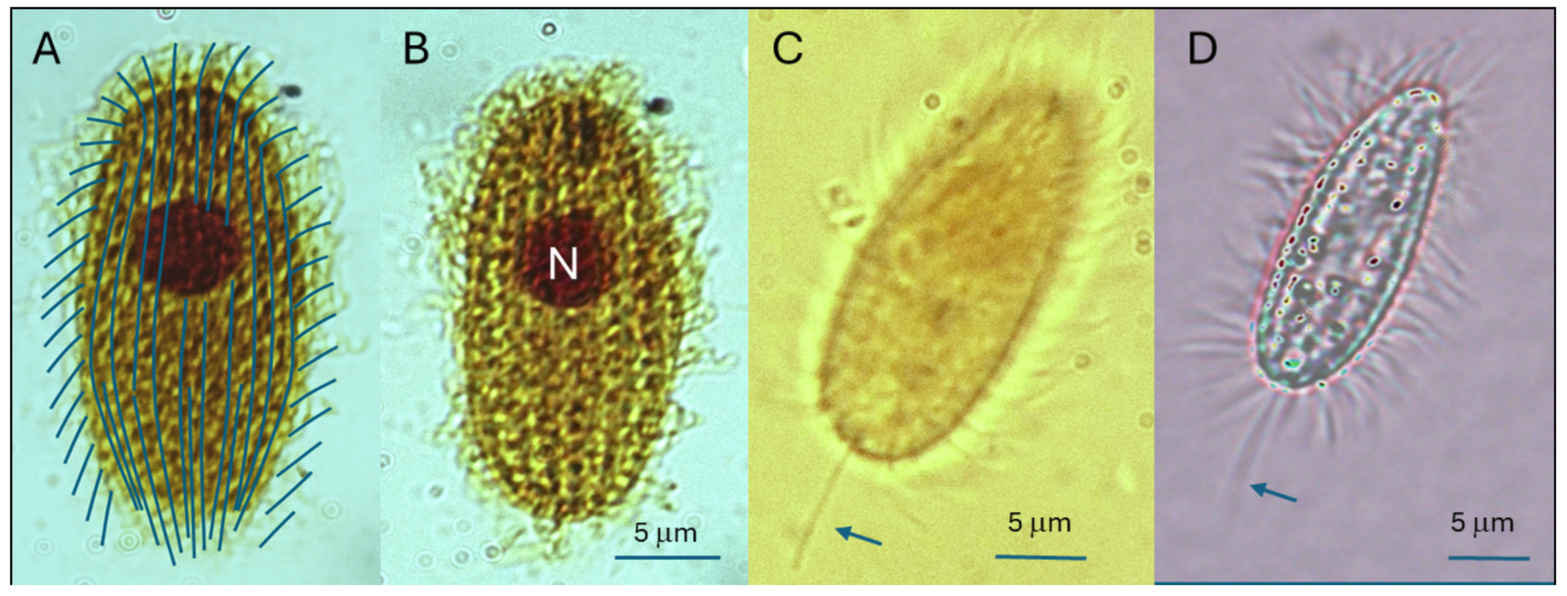
Somatic kineties and nuclear morphology of *U. marinum*. **(A)** Reconstruction of the somatic infraciliature showing longitudinal kineties distributed along the entire cell surface, with a characteristic curvature toward the anterior and posterior poles. **(B)** Live cell observed by bright-field microscopy showing the position and morphology of the macronucleus (N). **(C)** Phase-contrast micrograph of a live trophont highlighting the general cell outline and the posterior caudal cilium (cc). **(D)** Differential interference contrast (DIC) image of a live cell, providing enhanced visualization of cell shape and the caudal cilium (cc). Scale bars: 5 μm in **(A, B)**; 10 μm in **(C, D)**.

*Epithelioma papulosum cyprini* (EPC) cell monolayers were exposed for 24 h at 21 °C to *U. marinum* (5 × 103 cells mL^−1^), *P. dicentrarchi* (pathogenic control), *E. coli*, or *V. alginolyticus*. Cytopathic effects were examined microscopically (Figure 1). The ciliate density (5 × 103 cells mL^−1^) was selected based on preliminary observations indicating that this concentration ensured efficient predation on *Vibrio* cells while avoiding excessively high protozoan densities that could interfere with larval culture conditions. *Escherichia coli* was included in the assay as a non-pathogenic bacterial control to evaluate whether the presence of bacteria alone could induce cytopathic effects in the tested cell line. This control allowed discrimination between cytopathogenicity associated with the ciliate and potential effects caused by bacterial contamination or bacterial metabolites.

### 2.5 Fluorescent labeling of bacteria with FITC

To visualize bacterial ingestion by *Uronema marinum* and bivalve larvae, cultures of *V. splendidus* were fluorescently labeled with fluorescein isothiocyanate (FITC, Sigma-Aldrich, St. Louis, USA). Exponentially growing bacterial cells (OD_600_ = 0.6) were harvested by centrifugation (5,000 × g, 10 min, 4 °C) and washed twice with phosphate-buffered saline (PBS, pH 7.4). The pellet was resuspended in 1 mL of 0.1 mg mL^−1^ FITC solution prepared in carbonate–bicarbonate buffer (0.05 M, pH 9.5) and incubated for 30 min at room temperature in the dark with gentle agitation. After labeling, bacteria were washed three times with sterile seawater to remove unbound dye and resuspended to the desired concentration (10^6^ cells mL^−1^). FITC-labeled *V. splendidus* cells were then used in co-culture experiments with *U. marinum* trophozoites or with larvae of *V. corrugata* and *R. philippinarum*. Fluorescence images were obtained using an epifluorescence microscope equipped with a FITC filter set (excitation 495 nm; emission 520 nm). Labeled bacteria within ciliates or larvae were visualized as bright green, fluorescent signals.

### 2.6 Infection and protection assays in bivalve larvae

Larvae of *R. philippinarum* and *R. decussatus* (60–120 μm) were maintained at 21 °C in 24-well plates (1 mL seawater/well) and challenged with *V. alginolyticus* or *V. pectenicida* (10^6^ bacteria mL^−1^), with or without *U. marinum* (5 × 103 ciliates mL^−1^) or gentamicin (200 μg mL^−1^). Viability was assessed by counting motile (swimming) larvae at 24–168 h.

### 2.7 Phylogenetic analysis

The 18S rRNA gene sequence obtained from the isolate was compared with sequences deposited in GenBank using BLASTn. Representative sequences of scuticociliates and related taxa were retrieved and aligned using ClustalW implemented in MEGA version X. Phylogenetic relationships were inferred using the maximum-likelihood method under the Tamura–Nei substitution model. Node support was evaluated by bootstrap analysis with 1,000 replicates. The phylogenetic tree was used to determine the taxonomic placement of the isolate within the *Uronema marinum* clade.

### 2.8 Statistical analysis

Data are expressed as mean ± SD. Significant differences among treatments were determined by ANOVA followed by Tukey–Kramer multiple comparison tests (*p* ≤ 0.05).

## 3 Results

### 3.1 Identification and non-cytopathogenic character of *Uronema marinum*

Trophonts of the *U. marinum* CLIT/1 strain exhibited the typical ovoid to fusiform morphology characteristic of the species (Figure 1). Cells showed a mean length of 26 ± 2.93 μm and a mean width of 13 ± 1.05 μm. The somatic ciliature consisted of approximately 12–14 longitudinal kineties distributed uniformly over the entire cell surface, extending uninterruptedly from the anterior to the posterior pole. These kineties displayed a slight curvature at both ends, generating the characteristic holotrichous pattern described for scuticociliates (Figure 1A).

The oral infraciliature was located in the anteroventral region and appeared reduced, comprising a short paroral membrane and a very small adoral membranelle, as typically reported for *Uronema* spp. A single ovoid macronucleus was observed in the mid-body region, slightly eccentric, and was readily distinguishable in live cells by bright-field microscopy (Figure 1B). Phase-contrast and differential interference contrast imaging further highlighted the overall cell outline and the presence of a posterior caudal cilium (cc), a diagnostic feature of the genus (Figures 1C, D).

Overall, the morphometric parameters, number and arrangement of somatic kineties, reduced oral infraciliature, and nuclear morphology of the CLIT/1 trophonts are fully consistent with the diagnostic criteria for *U. marinum* (Supplementary Table S1).

**Figure 2:**
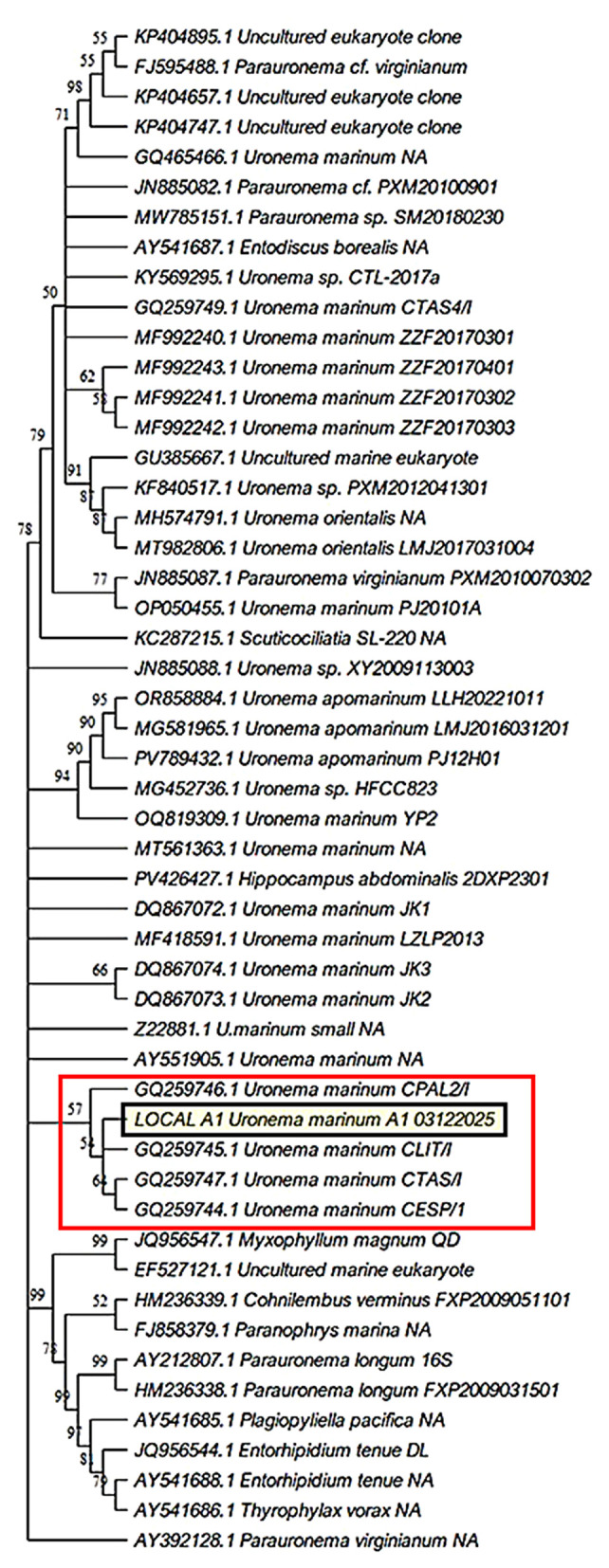
Phylogenetic position of the *Uronema marinum* isolate A1 inferred from 18S rRNA gene sequences. The maximum-likelihood tree was reconstructed using representative sequences of scuticociliates retrieved from GenBank. The isolate obtained in this study (*U. marinum* A1) is highlighted with a shaded background, and the clade containing this isolate is indicated by a box. Numbers at the nodes represent bootstrap support values (>50%) calculated from 1,000 replicates.

Molecular identification based on the 18S rRNA gene sequence confirmed the taxonomic assignment of the isolate. Phylogenetic analysis clustered the A1 isolate within the *U. marinum* clade with strong bootstrap support (Figure 2), showing 100% sequence identity with reference strain CLIT-1 (GenBank accession GQ259745.1).

**Figure 3:**
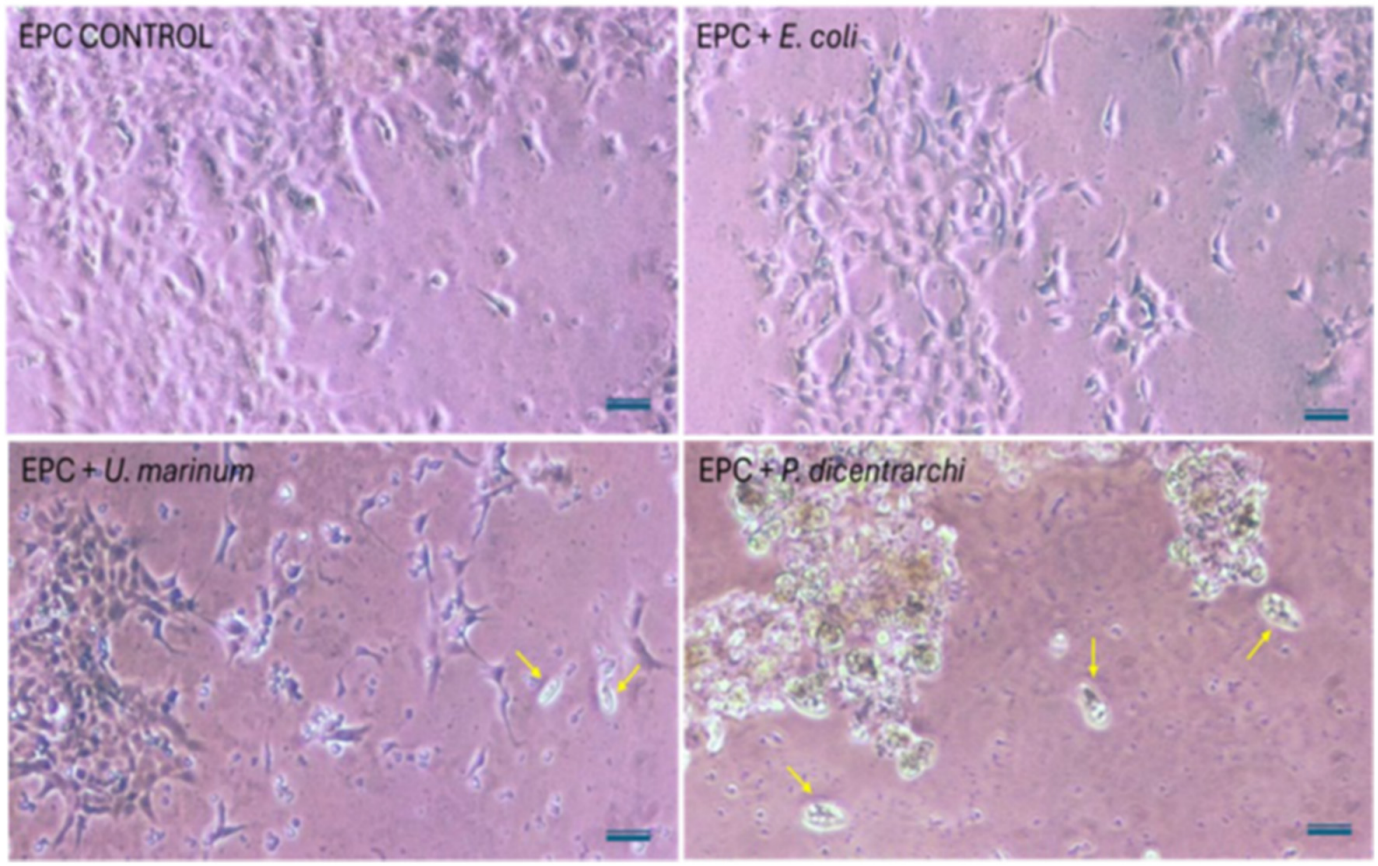
Cytopathic effect of *U. marinum* on EPC cell monolayers. *Epithelioma papulosum cyprini* (EPC) cell cultures were incubated for 24 h at 21 °C under different conditions: uninfected control (EPC CONTROL), exposure to *E. coli* (EPC + *E. coli*), exposure to *U. marinum* (EPC + *U. marinum*), and exposure to the pathogenic scuticociliate *P. dicentrarchi* (EPC + *P. dicentrarchi*). Yellow arrows indicate ciliates in the corresponding treatments. Scale bar = 50 μm.

To evaluate its cytopathogenic potential, EPC cell monolayers were exposed for 24 h to *U. marinum, Escherichia coli*, or the pathogenic scuticociliate *Philasterides dicentrarchi* as a positive control. As shown in Figure 3, EPC cells incubated with *U. marinum* or *E. coli* maintained normal morphology and monolayer integrity, whereas exposure to *P. dicentrarchi* resulted in extensive cell detachment and monolayer disruption. These results confirm that *U. marinum* CLIT/1 behaves as a non-pathogenic, strictly bacteriovorous ciliate under the experimental conditions tested. The control condition using *E. coli* did not induce detectable cytopathic effects, confirming that the cellular damage observed in the experimental groups was specifically associated with the ciliate.

The different interaction scenarios evaluated in this study are summarized in Supplementary Table S2.

### 3.2 Morphological observations of clam D-larvae under different experimental conditions

**Figure 4:**
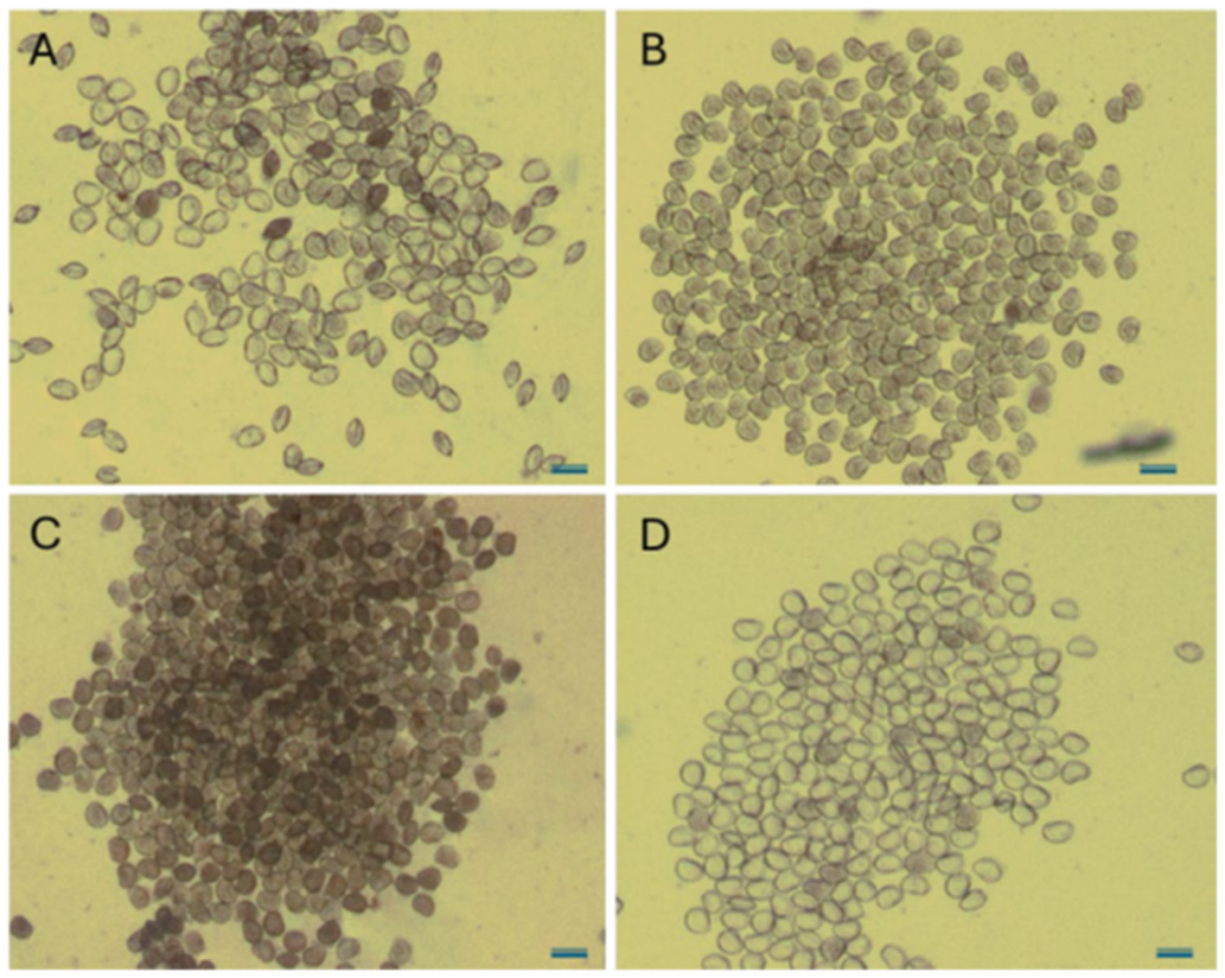
Light micrographs of D-larvae (early veliger stage) of the Japanese clam *Ruditapes philippinarum* under different experimental conditions. **(A)** Non-infected control; **(B)** larvae incubated with the bacteriovorous ciliate *U. marinum*; **(C)** larvae incubated with the pathogenic bacterium *V. alginolyticus*; and **(D)** larvae incubated with the scuticociliate *P. dicentrarchi*. Scale bar = 100 μm.

To evaluate the cytopathogenic effects of bacterial and ciliate exposure, *R. philippinarum* D-larvae (early veliger stage, ~60 μm) were examined by light microscopy under different experimental conditions (Figure 4). Non-infected control larvae (Figure 4A) displayed normal morphology, with intact bivalve shells, well-defined larval contours, homogeneous cytoplasmic appearance, and compact internal tissues. Larvae incubated with the bacteriovorous ciliate *U. marinum* (Figure 4B) were indistinguishable from controls, maintaining normal tissue organization and cytoplasmic integrity, with no evidence of vacuolization, cytolysis, or shell alteration, confirming the absence of cytopathogenic effects.

In contrast, exposure to the pathogenic bacterium *V. alginolyticus* (Figure 4C) induced marked cytopathological alterations. Affected larvae exhibited an increased density of granular material within the cytoplasm, suggestive of cytoplasmic condensation and tissue degeneration, together with loss of normal internal organization. These alterations are consistent with the progressive degradation of larval tissues commonly associated with *Vibrio*-mediated infections in bivalve larvae. Similarly, larvae exposed to the scuticociliate *Philasterides dicentrarchi* (Figure 4D) showed severe cytolytic damage, characterized by extensive vacuolization and an almost complete loss of internal cellular content, giving rise to a “ghost-like” or apparently empty appearance within intact shells. This pattern is indicative of rapid tissue digestion and strong histophagous activity.

Altogether, these microscopic observations demonstrate that *U. marinum* does not exert deleterious effects on clam larvae, whereas *V. alginolyticus* and *P. dicentrarchi* induce pronounced and distinct forms of structural damage. These findings further support the biosafety of *U. marinum* and reinforce its suitability as a non-pathogenic biological control agent in bivalve hatchery systems.

### 3.3 Bacterial ingestion by clam larvae provides a mechanistic basis for ciliate-mediated protection

**Figure 5:**
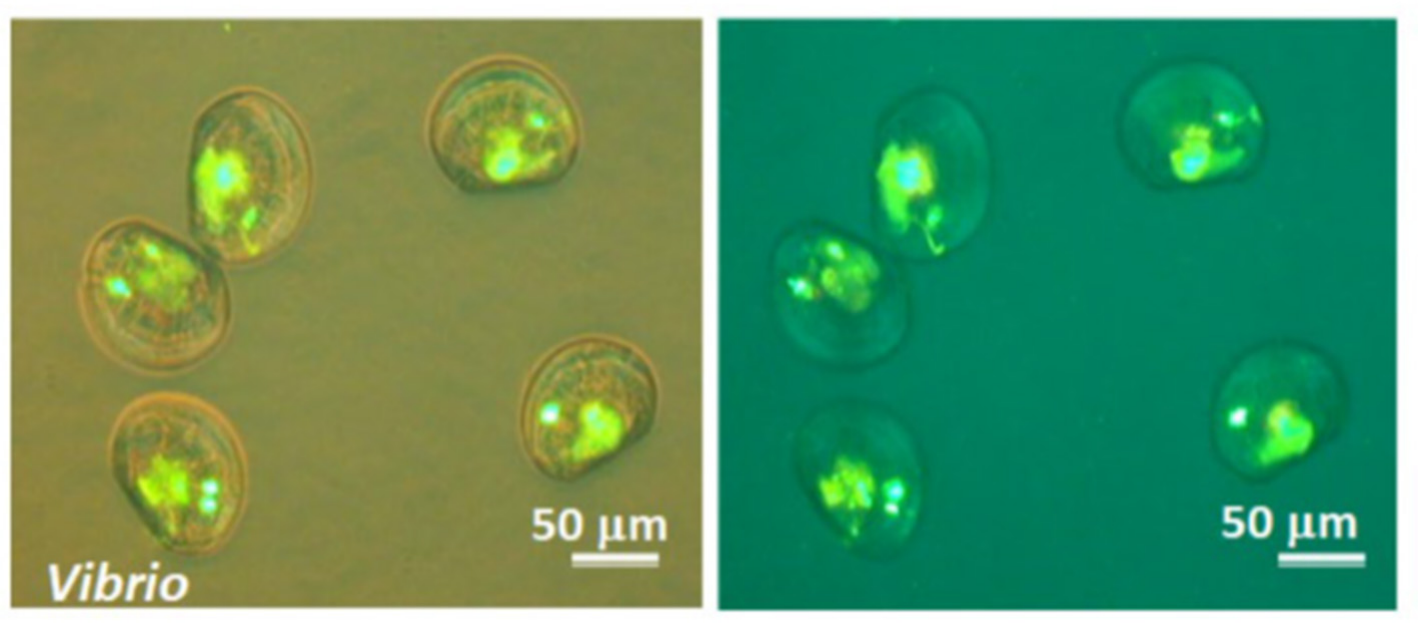
Fluorescence micrographs of clam larvae (*Venerupis corrugata*) ingesting FITC-labeled pathogenic bacteria. Larvae of *V. corrugata* were exposed to *V. splendidus* cells labeled with fluorescein isothiocyanate (FITC). Images show merged bright-field and fluorescence views **(left)** and fluorescence alone **(right)**. Scale bar = 50 μm.

Fluorescence microscopy confirmed the active ingestion of pathogenic bacteria by clam larvae (*V. corrugata*). After exposure to *V. splendidus* cells labeled with fluorescein isothiocyanate (FITC), intense green fluorescence was consistently detected within the digestive tract of the larvae (Figure 5). Merged bright-field and fluorescence images showed that the fluorescent signal was confined to the gut lumen, indicating true internalization of bacterial cells rather than surface attachment. Fluorescence-only images further highlighted the accumulation of FITC-labeled bacteria within the larval digestive system, consistent with active feeding and uptake of bacterial prey.

Importantly, the ingestion of *V. splendidus* by the larvae demonstrates that bacterial cells remain readily available for entry into the host under the experimental conditions used in the infection assays. This observation provides a mechanistic framework for the subsequent protection experiments, in which the presence of the bacteriovorous ciliate *U. marinum* markedly reduced larval mortality. By actively grazing on free-living bacterial cells, *U. marinum* is expected to decrease the number of pathogenic bacteria accessible for larval ingestion, thereby limiting bacterial entry into the digestive tract and reducing infection pressure.

### 3.4 Selective bacteriovory of *U. marinum* in the presence of bacteria and microalgae

**Figure 6:**
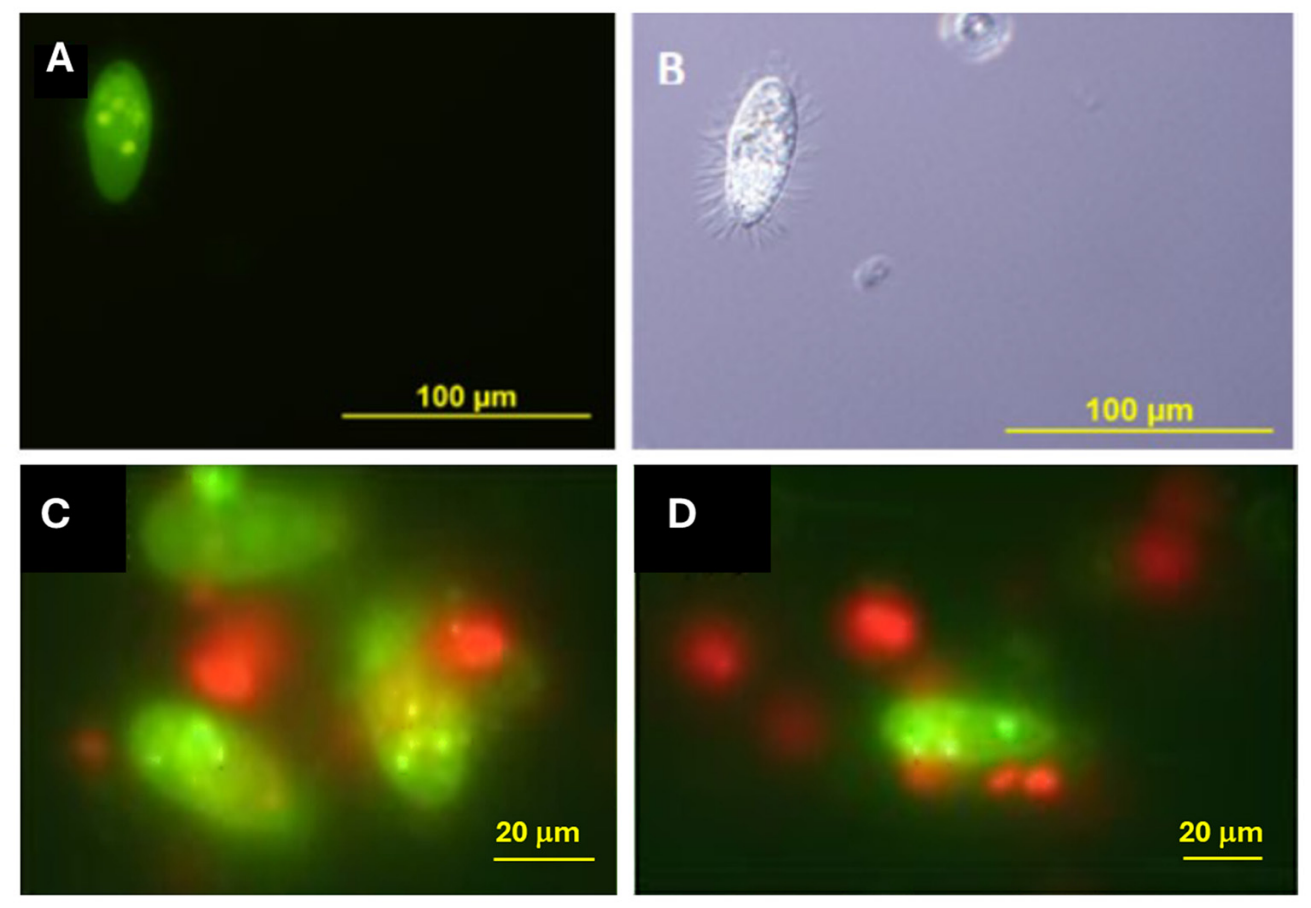
Selective bacteriovorous activity of *U. marinum* revealed by fluorescence and Nomarski microscopy during co-incubation with bacteria and microalgae. **(A)** Fluorescence micrograph showing *U. marinum* trophozoites after ingestion of FITC-labeled *Escherichia coli* DH5α. Green fluorescence corresponds to internalized bacterial cells, indicating active bacteriovory. **(B)** Differential interference contrast (Nomarski) micrograph of the same ciliate species, highlighting cell morphology and feeding activity. **(C, D)** Fluorescence micrographs of *U. marinum* co-incubated with FITC-labeled *E. coli* DH5α and the microalga *Isochrysis galbana*. Scale bars: 100 μm in **(A, B)**; 20 μm in **(C, D)**.

Fluorescence and Nomarski microscopy analyses revealed the selective bacteriovorous behavior of *U. marinum* during co-incubation with bacterial prey and microalgal cells (Figure 6). When exposed to FITC-labeled bacteria, intense green fluorescence was detected within *U. marinum* trophozoites (Figure 6A), indicating active ingestion and intracellular accumulation of bacterial cells. The presence of multiple discrete fluorescent signals within individual ciliates was consistent with the formation of food vacuoles containing bacterial aggregates.

Differential interference contrast (Nomarski) microscopy of the same specimens confirmed the typical morphology of actively feeding trophozoites (Figure 6B), supporting that fluorescence signals observed in panel A corresponded to intracellular bacterial uptake rather than external adhesion. These observations provide direct morphological and functional evidence of bacteriovory by *U. marinum*.

In co-incubation assays including both FITC-labeled bacteria and the microalga *I. galbana*, red/orange autofluorescent algal cells were clearly visible in the surrounding medium (Figures 6C, D). However, algal cells were not observed inside *U. marinum* trophozoites, whereas FITC-labeled bacteria were consistently detected in close association with, and internalized by, the ciliate. This clear spatial segregation between bacterial and microalgal fluorescence signals demonstrates that *U. marinum* selectively ingests bacterial prey and does not phagocytose microalgal cells under the experimental conditions tested.

Together, these results demonstrate that *U. marinum* maintains a strictly bacteriovorous trophic strategy even in the presence of abundant microalgae. This selective feeding behavior supports its ecological role as an efficient bacterial grazer and provides a mechanistic basis for its protective effect in subsequent infection assays, as bacterial pathogens are preferentially removed from the environment without interference with the microalgal component of bivalve larval diets.

During microscopic examination, none of the observed *U. marinum* trophozoites contained microalgal cells in their food vacuoles (0/200 ciliates examined), supporting the selective bacteriovorous feeding behavior of this species.

### 3.5 Growth of *U. marinum* with different *Vibrio* species

**Figure 7:**
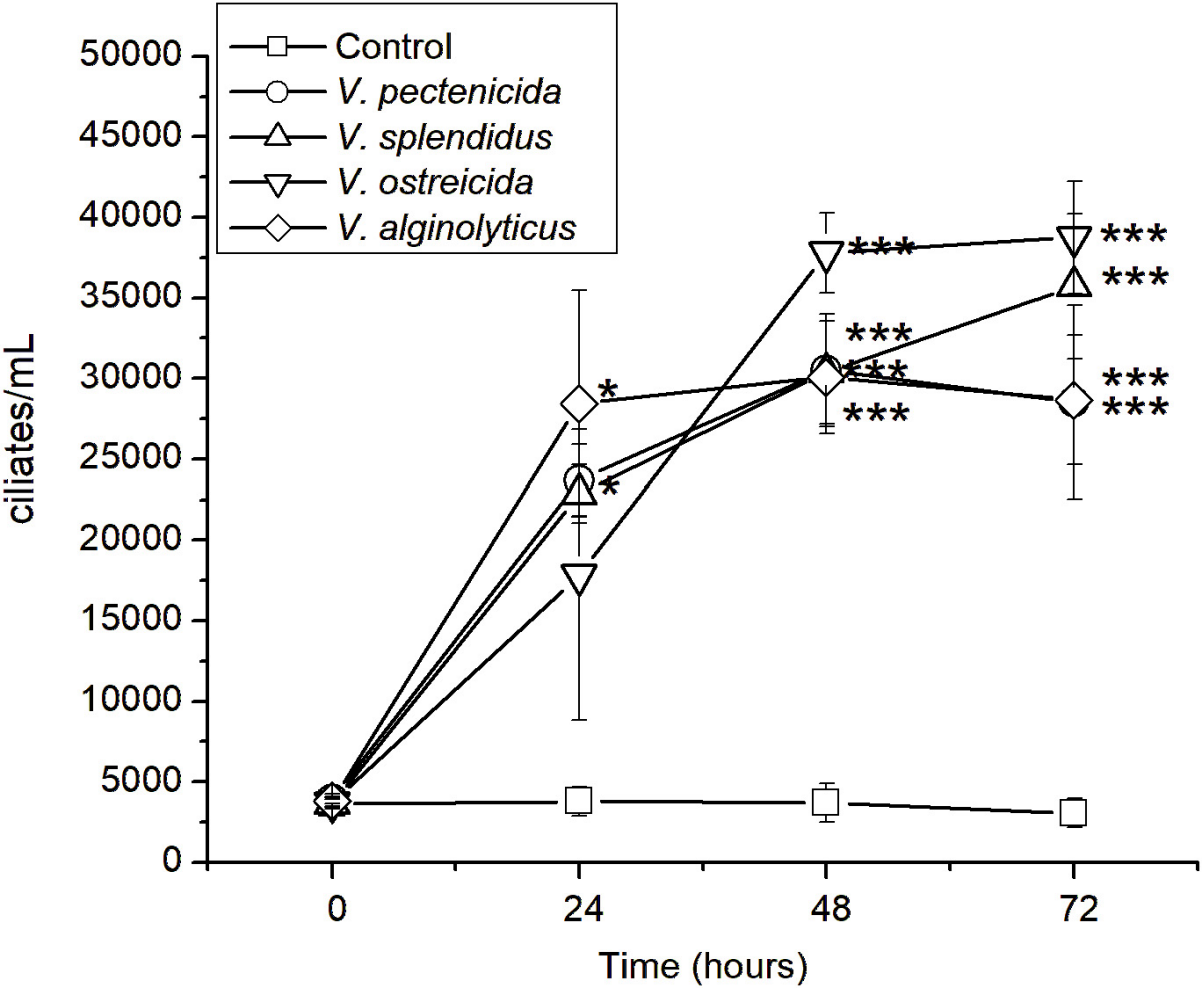
Growth dynamics of the bacteriovorous ciliate *U. marinum* in the presence of different *Vibrio* species. Ciliate proliferation was monitored over 72 h in monoxenic cultures containing distinct bacterial prey: *V. pectenicida, V. splendidus, V. ostreicida*, and *V. alginolyticus*. Ciliate density (cells mL^−1^) was quantified at 0, 24, 48, and 72 h. Data are expressed as mean ± standard deviation (*n* = 3). Asterisks indicate statistically significant differences compared with the control (*p* < 0.05, *p* < 0.01, *p* < 0.001).

The capacity of *U. marinum* to utilize various *Vibrio* species as bacterial prey was investigated in monoxenic cultures. As shown in Figure 7, ciliate populations increased markedly in the presence of all tested *Vibrio* strains (*V. pectenicida, V. splendidus, V. ostreicida*, and *V. alginolyticus*), whereas no growth was observed in control cultures without bacteria. After 24 h, ciliates reached densities between 2 × 10^4^ and 3 × 10^4^ cells mL^−1^ (*p* < 0.05), with *V. ostreicida* supporting the highest proliferation rate (>4 × 10^4^ ciliates mL^−1^ at 72 h; *p* < 0.001). These results indicate that *U. marinum* can feed efficiently on a wide spectrum of *Vibrio* species associated with bivalve diseases.

### 3.6 Growth of *U. marinum* fed with active and heat-inactivated *V. neptunius*

**Figure 8:**
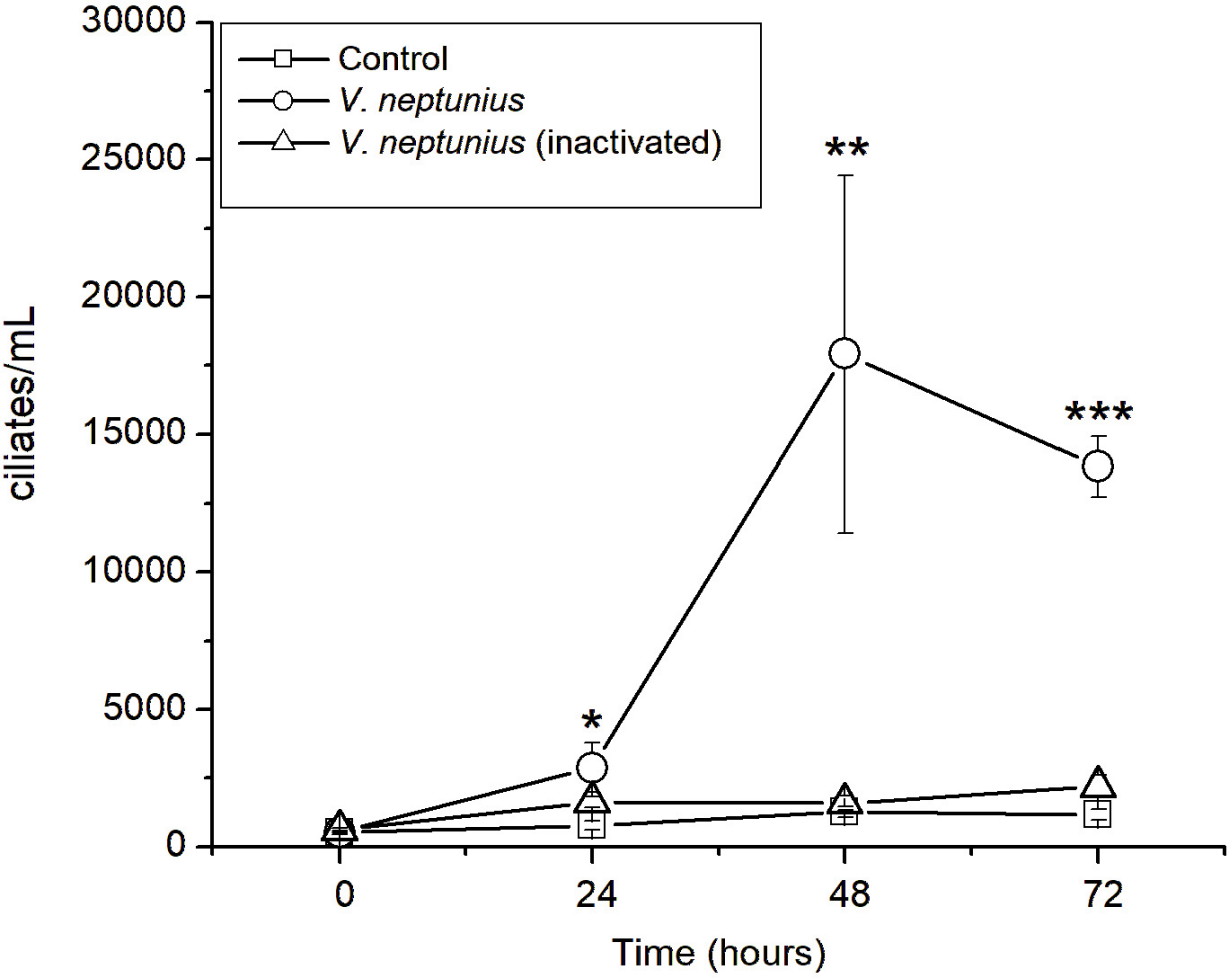
Growth assay of the bacteriovorous ciliate *U. marinum* fed with active and heat-inactivated *V. neptunius*. Ciliate proliferation was monitored over 72 h in cultures containing either live (active) or heat-inactivated (1 h at 60 °C) *V. neptunius* as the bacterial food source. The initial inoculum consisted of 625 ciliates mL^−1^ and 10^7^ bacterial cells mL^−1^. Data represent mean ± standard deviation (*n* = 3). Asterisks denote statistically significant differences compared with the control (*p* < 0.05, *p* < 0.01, *p* < 0.001).

To determine whether bacterial viability affects feeding and growth, *U. marinum* cultures were supplied with either live (active) or heat-inactivated (60 °C*, 1 h*) *V. neptunius*. As shown in Figure 8, ciliates fed with active *V. neptunius* exhibited strong proliferation, reaching approximately 2.5 × 10^4^ cells mL^−1^ at 48 h (*p* < 0.01). In contrast, cultures containing heat-inactivated bacteria or no bacterial prey showed negligible growth. These findings demonstrate that *U. marinum* requires metabolically active bacteria to sustain growth, suggesting that prey motility and physiological activity play key roles in ciliate feeding efficiency.

### 3.7 Protective effect of *U. marinum* on bivalve larvae challenged with bacterial and ciliate pathogens

**Figure 9:**
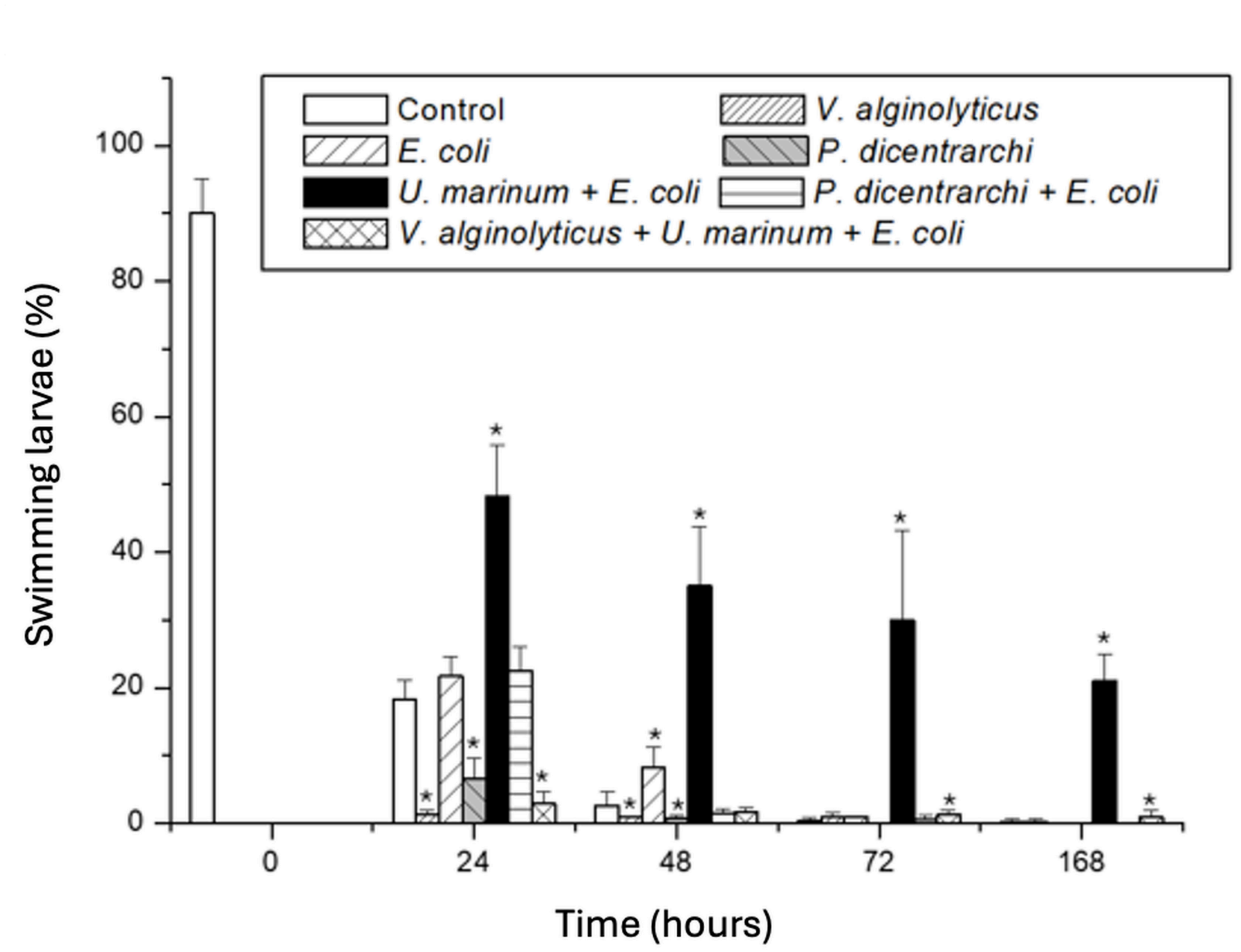
Protective effect of the ciliate *Uronema marinum* on the survival of bivalve larvae challenged with bacterial and ciliate pathogens. Larvae of the Japanese clam (*Ruditapes philippinarum*, 60 μm) were incubated *in vitro* at 21 °C with different microorganisms: *E. coli, V. alginolyticus, P. dicentrarchi*, or *U. marinum* alone or in combination, as indicated in the legend. Larval viability was determined as the percentage of swimming larvae (%) at various time points (0–168 h). Bars represent mean ± standard deviation of triplicate assays. Asterisks indicate statistically significant differences (*p* < 0.05) compared with the control group.

To evaluate the protective potential of *U. marinum*, larvae of *R. philippinarum* were experimentally challenged with *V. alginolyticus, P. dicentrarchi*, or *E. coli* under different combinations, with or without the ciliate. As shown in Figure 9, larval survival decreased sharply in groups exposed to *V. alginolyticus* or *P. dicentrarchi*, whereas co-incubation with *U. marinum* significantly improved survival (*p* < 0.05) throughout 168 h of incubation. The presence of *U. marinum* maintained the percentage of swimming larvae close to control levels, comparable to those obtained with the antibiotic gentamicin. Microscopic examination further confirmed these findings (Figure 4). Uninfected control larvae and those incubated with *U. marinum* displayed normal morphology and homogeneous cytoplasmic appearance, whereas those exposed to *V. alginolyticus* or *P. dicentrarchi* showed extensive cellular degradation and vacuolization, indicative of infection and tissue lysis. In contrast to the experimental treatments, the *E. coli* control did not produce significant cytopathic alterations, confirming that the observed effects were not attributable to the mere presence of bacteria in the culture system.

### 3.8 Protective effect of *U. marinum* on clam larvae challenged with different concentrations of *V. alginolyticus*

**Figure 10:**
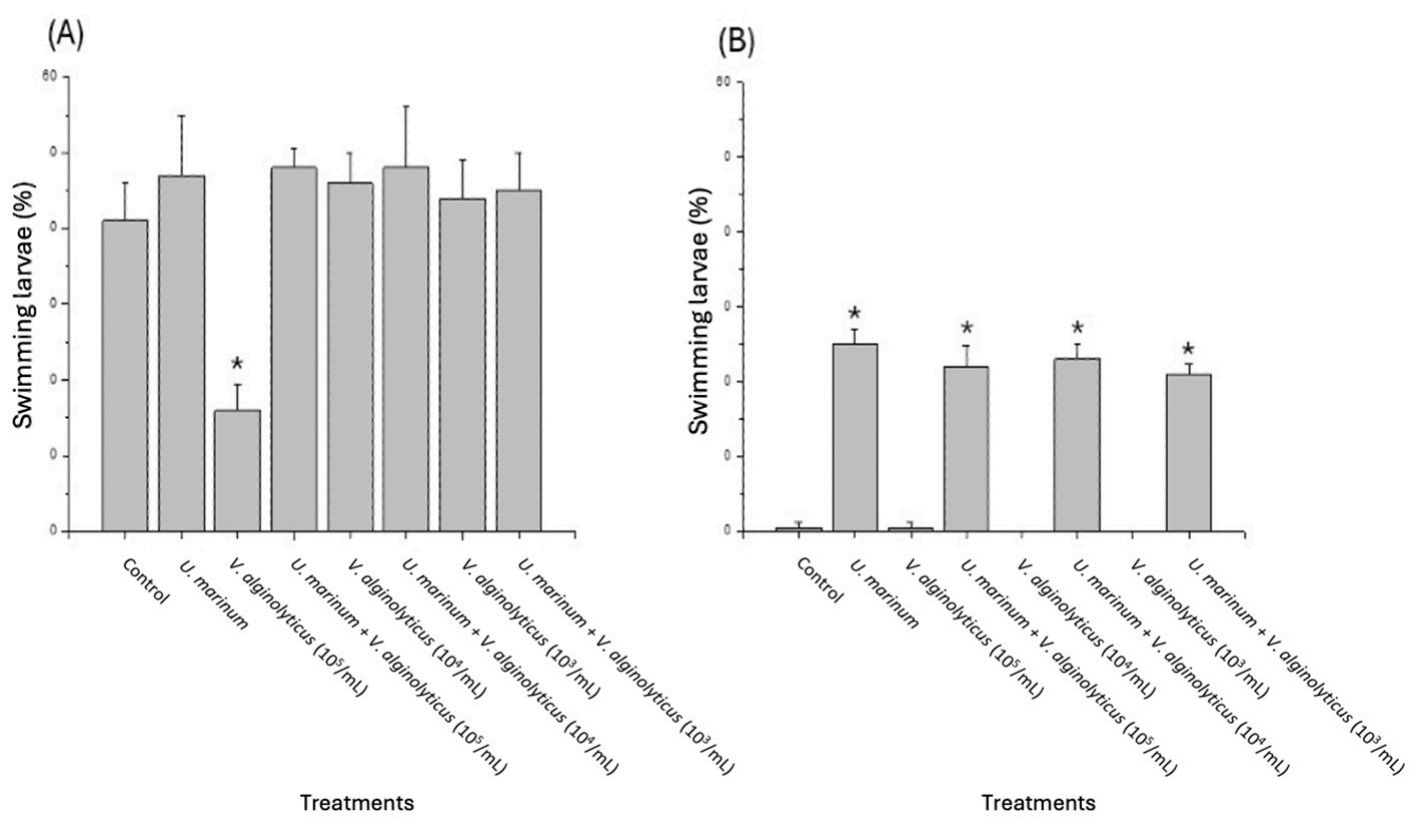
Protective effect of the ciliate *Uronema marinum* on clam larvae infected with different concentrations of the pathogenic bacterium *V. alginolyticus*. Larvae of **(A)** the fine clam *Ruditapes decussatus* and **(B)** the Japanese clam *R. philippinarum* were incubated *in vitro* at 21 °C with or without *U. marinum* (5 × 103 ciliates mL^−1^) and challenged with *V. alginolyticus* at different bacterial concentrations (103−10^5^ CFU mL^−1^). Larval viability was expressed as the percentage of swimming larvae after 72 h of incubation. Bars represent mean ± standard deviation of triplicate experiments. Asterisks indicate statistically significant differences (*p* < 0.05) compared with the control group.

The protective role of the bacteriovorous ciliate *U. marinum* was evaluated in larvae of the fine clam *R. decussatus* and the Japanese clam *R. philippinarum* experimentally challenged with increasing concentrations of the pathogenic bacterium *V. alginolyticus* (103−10^5^ CFU mL^−1^) (Figure 10). After 72 h of incubation at 21 °C, a clear dose-dependent reduction in larval viability was observed in the absence of the ciliate, with survival decreasing sharply at bacterial concentrations above 10^4^ CFU mL^−1^. In contrast, the presence of *U. marinum* (5 × 103 ciliates mL^−1^) significantly improved larval survival across all infection levels (*p* < 0.05). In both *R. decussatus* and *R. philippinarum*, the percentage of swimming larvae remained close to control values even at the highest bacterial dose, indicating an effective protective action of the ciliate. The magnitude of protection was consistent between the two clam species, demonstrating that *U. marinum* exerts a broad-spectrum bioprotective effect against *Vibrio* infections independent of host species or pathogen concentration. These results confirm that *U. marinum* can substantially mitigate the cytopathogenic effects of *V. alginolyticus* on bivalve larvae by controlling bacterial density through active bacteriovory. This supports its potential application as a natural biological control agent in hatchery systems to prevent *Vibrio*-related mortalities without the use of antibiotics.

### 3.9 Dose-dependent protection and antibiotic comparison

**Figure 11:**
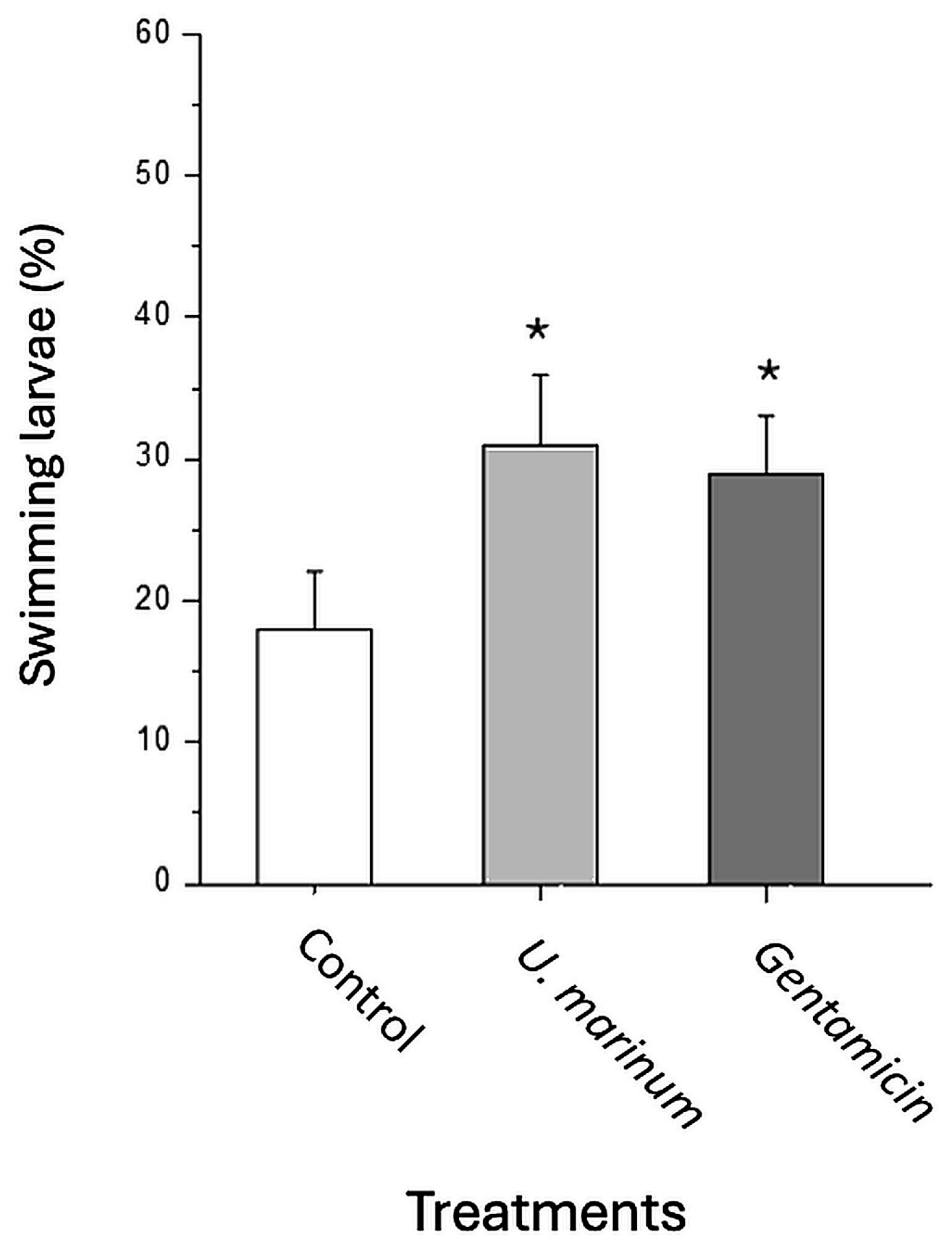
Comparative protective effects of *U. marinum* and gentamicin on clam larvae exposed to high bacterial loads. Larvae of the fine clam *R. decussatus* (90 μm) obtained from hatchery tanks exhibiting elevated bacterial concentrations (2 × 10^6^ bacteria mL^−1^) and high mortality rates were incubated *in vitro* for 72 h at 21 °C with *U. marinum* (5 × 103 ciliates mL^−1^) or the broad-spectrum antibiotic gentamicin (200 μg mL^−1^). Bars represent mean ± standard deviation of triplicate assays. Asterisks indicate statistically significant differences (*p* < 0.05) vs. control.

The effect of *U. marinum* on larval protection was further assessed at different bacterial concentrations. As shown in Figure 11, both *R. philippinarum* and *R. decussatus* larvae exhibited significantly higher survival rates in the presence of *U. marinum* (*p* < 0.05) across *V. alginolyticus* concentrations ranging from 103 to 10^5^ CFU mL^−1^, confirming a dose-independent protective effect. When larvae from hatchery tanks with high bacterial loads (2 × 10^6^ CFU mL^−1^) were treated with either *U. marinum* or gentamicin, both treatments significantly enhanced larval survival compared with untreated controls (Figure 11). The magnitude of protection conferred by *U. marinum* was comparable to that achieved with gentamicin, supporting its potential as a natural biocontrol alternative to antibiotics.

## 4 Discussion

### 4.1 Non-cytopathogenic character and taxonomic confirmation of *U. marinum*

This study provides an integrated morphological, molecular, and biological assessment of *U. marinum*, allowing its taxonomic identity and non-cytopathogenic behavior to be critically evaluated in the context of its potential application in aquaculture. Morphological and molecular analyses confirmed that the isolated ciliate corresponded to *U, marinum*, a free-living scuticociliate previously reported in coastal marine habitats worldwide (Dragesco and Dragesco-Kernéis, 1986; Song et al., 2009). In addition to its typical ovoid–fusiform shape, the trophonts displayed a posterior caudal cilium, a diagnostic morphological feature of the genus *Uronema* that was consistently observed in live cells. The 18S rRNA sequence (100% identity with strain CLIT-1, GenBank GQ259745.1) further verified its taxonomic identity. Cytopathogenicity assays demonstrated that *U. marinum* was completely non-pathogenic to EPC fish cells, in sharp contrast to the severe lytic effects caused by *Philasterides dicentrarchi*, a histophagous ciliate responsible for scuticociliatosis in fish (Iglesias et al., 2001; Paramá et al., 2006). The absence of cytopathic alterations in EPC monolayers and in clam larvae exposed to *U. marinum* unequivocally demonstrates its biosafety and non-parasitic nature, distinguishing it from closely related pathogenic scuticociliates. This feature is essential to validate its use in aquaculture as a potential biological control agent.

### 4.2 Lack of cytopathogenic effects on clam larvae

Building on the morphological and biological characterization of *U. marinum*, its interaction with bivalve larvae was subsequently evaluated to determine whether this ciliate exerts any cytopathogenic effects under experimental conditions.

Microscopic observations of *R. philippinarum* D-larvae confirmed that *U. marinum* does not cause any morphological or cytological damage, in contrast to the extensive tissue degradation induced by *V. alginolyticus* and *P. dicentrarchi*. These findings are consistent with previous reports of *Vibrio*- and scuticociliate-induced pathology in hatchery larvae (Prado et al., 2014; Dubert et al., 2017). The maintenance of structural integrity in larvae co-incubated with *U. marinum* further confirms that its ecological role is strictly bacteriovorous and that it is safe for bivalve larvae, even under prolonged exposure. This biosafety profile distinguishes *U. marinum* from opportunistic ciliates occasionally associated with larval mortality events.

### 4.3 Evidence of active bacteriovory and trophic interactions

Having established the absence of cytopathogenic effects of *U. marinum* on bivalve larvae, we next examined whether its interaction with larvae involved active trophic processes. Fluorescence and Nomarski microscopy provided direct evidence that *U. marinum* is a strictly bacteriovorous ciliate capable of actively ingesting and digesting pathogenic bacteria.

The detection of FITC-labeled *V. splendidus* within both *V. corrugata* larvae and *U. marinum* trophozoites demonstrates that bacterial cells are readily internalized by susceptible larvae and simultaneously constitute suitable prey for the ciliate. In larvae, fluorescence was confined to the digestive tract, indicating uptake through normal feeding mechanisms. In contrast, in *U. marinum*, multiple intracellular fluorescent signals were consistent with phagocytosis and food vacuole formation. Together, these observations provide clear evidence of active bacteriovory and bacterial digestion.

Importantly, co-incubation experiments including the microalga *I. galbana* revealed pronounced trophic selectivity. While algal cells were abundant in the surrounding medium and easily detected by their red/orange autofluorescence, they were never observed inside *U. marinum* trophozoites. In contrast, FITC-labeled bacteria were consistently internalized, indicating that *U. marinum* selectively targets bacterial prey and does not behave as a generalist phagotroph. This behavior is consistent with the ecological role of many marine scuticociliates as specialized bacterial grazers within microbial food webs (Fenchel, 1987; Sherr and Sherr, 2002).

From an applied perspective, this selective bacteriovory provides a mechanistic basis for the protective effects observed in subsequent larval challenge assays. By actively grazing on free-living pathogenic *Vibrio* cells while leaving microalgal components of the diet unaffected, *U. marinum* is expected to reduce the number of infectious units available for larval ingestion, thereby lowering infection pressure. This bacteriovory-driven regulation of bacterial density represents a fundamental ecological process (Sherr and Sherr, 2002) that can be exploited for sustainable disease control in hatchery systems. It also directly links the trophic observations reported here with the enhanced larval survival described in the following sections.

Although our results demonstrate efficient bacteriovory by *U. marinum* toward pathogenic *Vibrio* species, the potential feeding preferences of this ciliate among different bacterial taxa were not investigated in detail. In microbial predator–prey systems, protozoan grazers often display prey selectivity and density-dependent functional responses that can strongly influence bacterial community composition. Future studies should therefore examine prey selectivity of *U. marinum* under more complex microbial assemblages to better understand its ecological role and its potential application in microbial management strategies in aquaculture systems.

### 4.4 Growth of *U. marinum* with multiple *Vibrio* species

The rapid proliferation of *U. marinum* in monoxenic cultures containing *V. splendidus, V. pectenicida, V. ostreicida*, and *V. alginolyticus* confirms its ability to exploit a wide spectrum of bacterial prey. This trophic versatility is consistent with the ecological role of scuticociliates as major bacterial grazers in coastal waters (Fenchel, 1987; Song et al., 2009). The particularly high growth rate obtained with *V. ostreicida*—a virulent species in clam hatcheries (Prado et al., 2014)—suggests that *U. marinum* could naturally help suppress harmful bacterial blooms. Such behavior reflects the functional adaptability of the ciliate to the microbial composition of aquaculture environments.

### 4.5 Dependence on bacterial viability for ciliate growth

The inability of *U. marinum* to grow on heat-inactivated *V. neptunius* demonstrates that prey viability is critical for its nutrition. Similar selectivity has been described in marine ciliates that rely on the motility or physiological activity of live bacteria for recognition and ingestion (Fenchel, 1987; Sherr and Sherr, 2002). This finding supports that *U. marinum* interacts ecologically with metabolically active bacteria, reinforcing its role as a true predator within the microbial loop rather than a saprophytic scavenger.

### 4.6 Protective effects against bacterial and ciliate pathogens

The experimental challenges demonstrated that *U. marinum* significantly enhances larval survival when co-incubated with *V. alginolyticus* or *P. dicentrarchi*. This effect can be attributed to its active predation on bacteria and possibly on ciliate pathogens, consistent with its known capacity to engulf a variety of microbial prey (Song et al., 2009; Sherr and Sherr, 2002). The discovery that *P. dicentrarchi* can cause high mortality in clam larvae expands its recognized host range beyond fish and emphasizes the need to distinguish pathogenic scuticociliates from benign bacteriovores in hatchery environments (Iglesias et al., 2001; Paramá et al., 2006). The coexistence of both types of ciliates in natural and artificial systems underscores the importance of accurate identification for microbial management strategies. Comparable bacteriovorous effects have been reported in other ciliates used experimentally against *Vibrio* infections (Sherr and Sherr, 2002).

### 4.7 Broad-spectrum protection across bacterial concentrations and host species

The observation that *U. marinum* maintained high larval survival across a wide range of *V. alginolyticus* concentrations (103−10^5^ CFU mL^−1^) and in two clam species (*R. philippinarum* and *R. decussatus*) demonstrates its broad-spectrum bioprotective capacity. These results indicate that the ciliate can adapt to variable microbial pressures, keeping pathogen levels below the infection threshold through continuous bacteriovory. This behavior aligns with the ecological concept of density-dependent regulation observed in microbial predator–prey systems (Sherr and Sherr, 2002). Such versatility also suggests that *U. marinum* could be maintained as a stable component of hatchery microbiomes, providing ongoing protection without the need for continuous reintroduction.

### 4.8 Comparison with antibiotic treatment and implications for sustainable aquaculture

The finding that *U. marinum* provided a protective effect equivalent to gentamicin—one of the most common antibiotics used in hatcheries (Dubert et al., 2017)—is particularly significant. This demonstrates that biological regulation by ciliates can achieve similar efficacy without promoting antimicrobial resistance or disturbing beneficial microbiota (Cabello et al., 2013). Compared with other biocontrol strategies, such as probiotics (Pérez-Sánchez et al., 2018), quorum-quenching bacteria (Defoirdt et al., 2011), or bacteriophage therapy (Kalatzis et al., 2018), *U. marinum* operates through a distinct ecological mechanism: active predation. Rather than altering bacterial metabolism or signaling, it physically removes pathogens from the environment, reducing infection risk and stabilizing the microbial community. In practical terms, this trophic regulation mechanism could be applied in bivalve hatchery to naturally reduce *Vibrio* loads in larval rearing tanks, thereby contributing to microbiological stability and improved larval survival. This approach complements existing probiotic-based methods and aligns with the emerging concept of ecological engineering of hatchery microbiomes for disease prevention.

### 4.9 Ecological and biotechnological significance

Overall, this study provides the first experimental demonstration that a free-living ciliate can act as a biological control agent against *Vibrio*-induced infections in bivalve larvae. By combining non-pathogenicity, broad prey range, and ecological persistence, *U. marinum* represents a novel and environmentally safe strategy to improve larval survival in bivalve hatcheries. Importantly, the protective effect of *U. marinum* is mechanistically supported by direct experimental evidence of active and selective bacteriovory. Fluorescence and Nomarski microscopy demonstrated that *U. marinum* actively ingests and digests pathogenic *Vibrio* cells, while excluding non-bacterial particulate food such as microalgae, even under conditions where algal cells are abundant.

The trophic mechanism underlying this protection—direct bacteriovory—links fundamental microbial ecology with applied aquaculture biotechnology. By selectively removing free-living bacterial pathogens from the rearing environment, *U. marinum* reduces the pool of infectious units available for ingestion by larvae, thereby lowering infection pressure without interfering with the microalgal component of larval diets. Harnessing such predator–pathogen interactions can significantly reduce antibiotic dependence, contributing to microbiome-based disease management and One Health aquaculture frameworks (Llewellyn et al., 2014).

Although further validation at pilot-hatchery scale will be necessary to optimize inoculation strategies, assess long-term ecological stability, and confirm compatibility with beneficial microbiota, the results presented here highlight *U. marinum* as a promising prototype for next-generation biological control agents in sustainable aquaculture systems.

### 4.10 General implications

Altogether, this study demonstrates that the ecological role of a bacteriovorous ciliate can be directly exploited to protect bivalve larvae from bacterial disease. The combined evidence of (i) active ingestion of pathogenic *Vibrio* cells by larvae, (ii) selective bacteriovory by *U. marinum* in the presence of microalgae, and (iii) enhanced larval survival in infection assays supports a coherent mechanistic model in which microbial predation reduces pathogen availability prior to host entry.

By exploiting trophic interactions inherent to marine microbial ecosystems, this approach promotes antibiotic-free, ecologically balanced hatchery systems. The strict bacteriovorous behavior and trophic selectivity of *U. marinum* ensure that bacterial pathogens are targeted without disrupting beneficial microalgal–larval interactions, aligning this strategy with the goals of sustainable aquaculture and ecosystem-based disease management.

Although the present study demonstrates the strong bacteriovorous activity of *U. marinum* against several pathogenic *Vibrio* species, the potential effects of introducing this ciliate on the broader microbial community present in aquaculture systems were not specifically evaluated. In natural and hatchery environments, microbial communities include both pathogenic and beneficial bacteria that contribute to nutrient cycling, larval health, and overall system stability. Therefore, the introduction or enrichment of bacteriovorous protozoa could potentially influence the composition and dynamics of these microbial assemblages. Future studies should address this aspect by analyzing microbial community responses using culture-independent approaches, such as high-throughput sequencing or metagenomic analyses, to determine whether the presence of *U. marinum* selectively reduces pathogenic bacteria while preserving beneficial microbiota. Such studies would be essential to assess the ecological safety and practical feasibility of implementing this ciliate as a biological control agent in hatchery environments.

Overall, our results highlight the potential of bacteriovorous ciliates such as *U. marinum* as natural regulators of bacterial populations in aquaculture systems. Harnessing protozoan predation as a microbial management strategy could represent an environmentally friendly alternative to antibiotic use, contributing to the development of more sustainable and resilient hatchery practices.

## 5 Conclusions

1. *U. marinum* is a non-pathogenic, strictly bacteriovorous ciliate capable of actively ingesting and digesting pathogenic *Vibrio* species while excluding microalgal cells, demonstrating pronounced trophic selectivity.
2. The selective bacteriovory of *U. marinum* provides a clear mechanistic basis for its protective effect, as the ciliate reduces the availability of infectious bacterial cells for larval ingestion.
3. Application of *U. marinum* significantly enhances bivalve larval survival during *Vibrio* infections, providing protection comparable to antibiotic treatment without disrupting the microalgal component of larval diets.
4. The use of *U. marinum* represents a promising, sustainable biological alternative for bacterial control in bivalve hatcheries, grounded in fundamental predator–prey interactions within marine microbial ecosystems.

## Data Availability

The raw data supporting the conclusions of this article will be made available by the authors, without undue reservation.
