# Supplementary material for: The bacteriovorous ciliate Uronema marinum as a natural biological control agent against Vibrio infections in bivalve hatcheries: a sustainable alternative to antibiotics
Source: Front Microbiol. 2026 Apr 10;17:1806526. doi: 10.3389/fmicb.2026.1806526 (PMC13106148; doi:10.3389/fmicb.2026.1806526)
Supplement: Supplementary file 1 [file Table_1.docx]

**Supplementary Table S1.** Morphological and biological characteristics of the CLIT/1 isolate compared with the reference description of *Uronema marinum*.

| **Characteristic** | **A1 isolate (this study)** | **Type description of *Uronema marinum*** |
| --- | --- | --- |
| Cell shape | Fusiform to ovoid | Fusiform to ovoid |
| Cell size (µm) | 24–30 µm long; ~12–14 µm wide | 20–35 µm long; 10–18 µm wide |
| Somatic kineties | 12–14 longitudinal kineties | 12–15 longitudinal kineties |
| Arrangement of kineties | Holotrichous; continuous from anterior to posterior pole | Holotrichous; uninterrupted from anterior to posterior |
| Oral infraciliature | Reduced; short paroral membrane + minute adoral membranelle | Reduced; short paroral membrane + minute adoral membranelle |
| Macronucleus | Single, ovoid, centrally positioned | Single, ovoid; centrally to subcentrally positioned |
| Caudal cilium | Present in all trophonts; well defined | Variably expressed; sometimes faint or absent |
| Cytoplasm | Granular, homogeneous; no vacuolation | Granular; no abnormal inclusions |
| Motility | Rapid, erratic swimming typical of scuticociliates | Rapid, erratic swimming |
| Cytopathogenicity | Non-cytopathogenic to EPC cells | Free-living, non-parasitic; non-cytopathogenic |
| Feeding behavior | Strictly bacterivorous; ingestion of *Vibrio* spp. confirmed | Bacterivorous; feeds on free-living bacteria |
| 18S rRNA phylogeny | Clusters within the *U. marinum* clade | Defines the reference clade |
| Similarity to reference sequences | 100% identity with reference strains | Reference sequences defining species |
