# Supplementary material for: The bacteriovorous ciliate Uronema marinum as a natural biological control agent against Vibrio infections in bivalve hatcheries: a sustainable alternative to antibiotics
Source: Front Microbiol. 2026 Apr 10;17:1806526. doi: 10.3389/fmicb.2026.1806526 (PMC13106148; doi:10.3389/fmicb.2026.1806526)
Supplement: Supplementary file 2 [file Table_2.docx]

| **Experiment** | **Microorganisms involved** | **Host organism** | **Objective** | **Main outcome** |
| --- | --- | --- | --- | --- |
| Cytopathogenicity assay | *Philasterides dicentrarchi* | Fish cell line | Evaluate cytopathic effect | Extensive monolayer destruction and cell detachment |
| Cytopathogenicity control | *Uronema marinum* | Fish cell line | Evaluate potential cytotoxicity | No cytopathic effect observed |
| Cytopathogenicity control | *Escherichia coli* | Fish cell line | Non-pathogenic bacterial control | No cytopathic effect |
| Bacteriovory assay | *Uronema marinum* + *Vibrio* spp. | — | Assess bacteriovorous activity | Efficient consumption of *Vibrio* cells |
| Bacterial interaction assay | *Uronema marinum* + pathogenic *Vibrio* spp. | — | Evaluate reduction of bacterial abundance | Significant decrease in bacterial density |
| Larval protection assay | *Uronema marinum* + pathogenic *Vibrio* spp. | Bivalve larvae | Evaluate protective effect | Increased larval survival |
| Antibiotic comparison | Gentamicin + pathogenic *Vibrio* spp. | Bivalve larvae | Compare with conventional treatment | Survival improvement similar to ciliate treatment |

**Supplementary Table S2.** Summary of microbial interactions and host responses investigated in the experimental system. **Summary of experimental interactions between ciliates, bacterial species, and host organisms evaluated in this study.**
**Summary of experimental interactions between ciliates, bacterial species, and host organisms evaluated in this study.**
